# Supplementary material for: Major QTLs for Resistance to Early and Late Leaf Spot Diseases Are Identified on Chromosomes 3 and 5 in Peanut (Arachis hypogaea)
Source: Front Plant Sci. 2019 Jul 5;10:883. doi: 10.3389/fpls.2019.00883 (PMC6625158; doi:10.3389/fpls.2019.00883)
Supplement: Supplementary file 3 [file Presentation_1.pptx]

## Slide 1
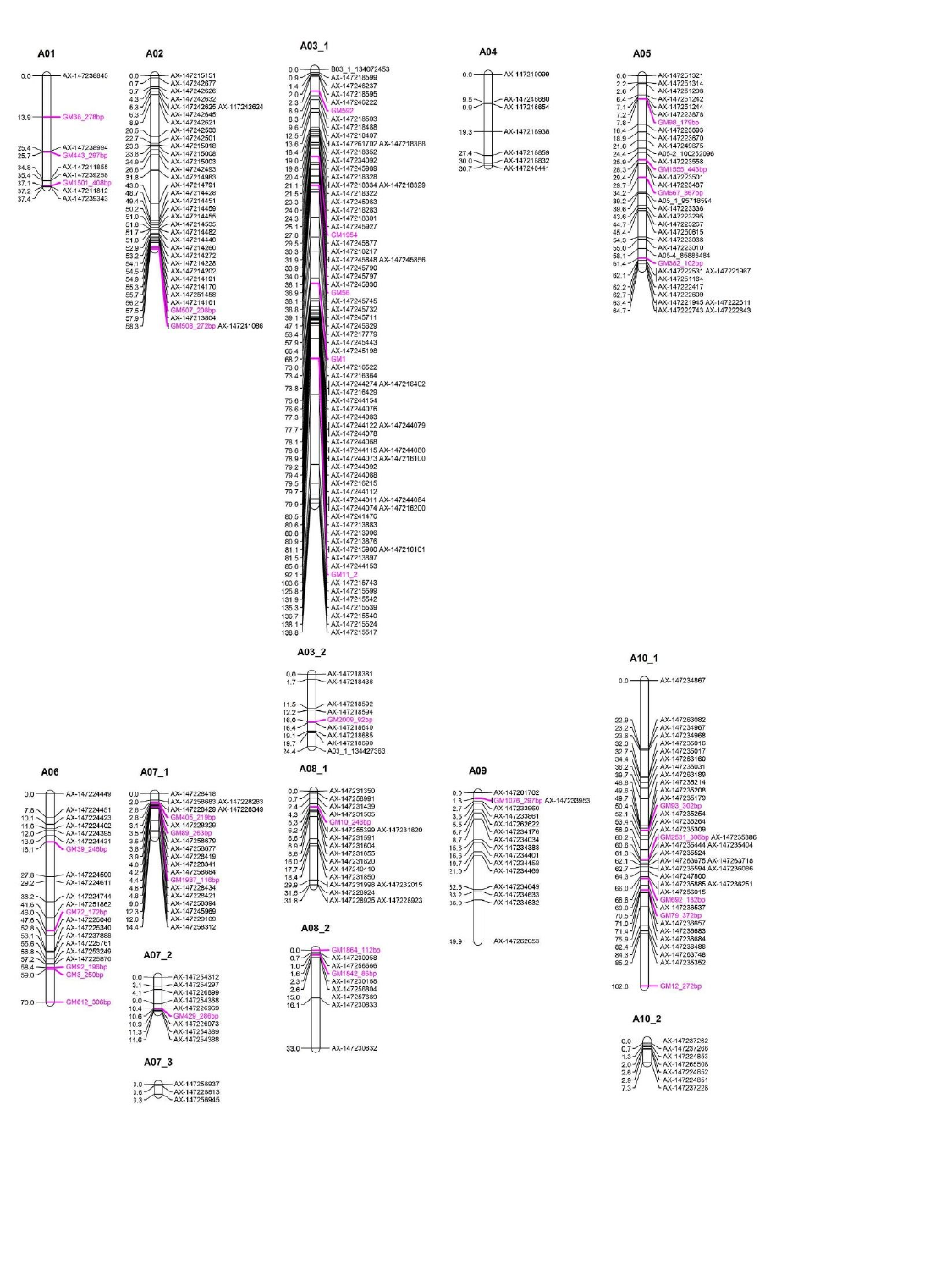

## Slide 2
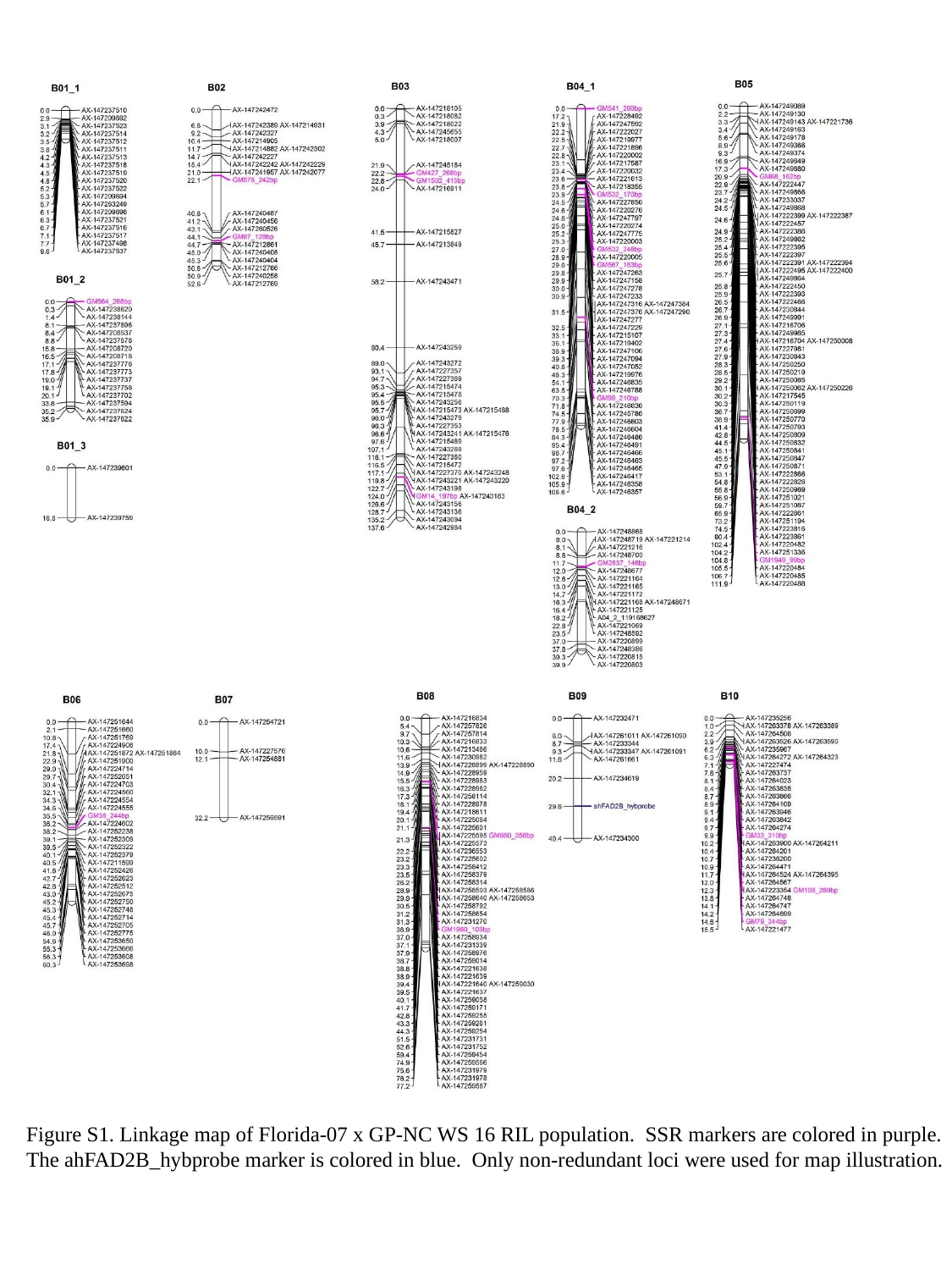

Figure S1. Linkage map of Florida-07 x GP-NC WS 16 RIL population. SSR markers are colored in purple.
The ahFAD2B_hybprobe marker is colored in blue. Only non-redundant loci were used for map illustration.
